# Structural Determinants of *Arabidopsis thaliana* Hyponastic Leaves 1 Function *In Vivo*

**DOI:** 10.1371/journal.pone.0113243

**Published:** 2014-11-19

**Authors:** Paula Burdisso, Fernando Milia, Arnaldo L. Schapire, Nicolás G. Bologna, Javier F. Palatnik, Rodolfo M. Rasia

**Affiliations:** 1 Instituto de Biología Molecular y Celular de Rosario, Rosario, Argentina; 2 Área Biofísica, Facultad de Ciencias Bioquímicas y Farmacéuticas, Universidad Nacional de Rosario, Rosario, Argentina; 3 Center for Research in Agricultural Genomics CRAG (CSIC-IRTA-UAB-UB), Edifici CRAG-Campus UAB, Bellaterra (Cerdanyola del Vallés), Barcelona, Spain; 4 Swiss Federal Institute of Technology (ETH), Zurich, Switzerland; Universidad Miguel Hernández de Elche, Spain

## Abstract

MicroRNAs have turned out to be important regulators of gene expression. These molecules originate from longer transcripts that are processed by ribonuclease III (RNAse III) enzymes. Dicer proteins are essential RNAse III enzymes that are involved in the generation of microRNAs (miRNAs) and other small RNAs. The correct function of Dicer relies on the participation of accessory dsRNA binding proteins, the exact function of which is not well-understood so far. In plants, the double stranded RNA binding protein Hyponastic Leaves 1 (HYL1) helps Dicer Like protein (DCL1) to achieve an efficient and precise excision of the miRNAs from their primary precursors. Here we dissected the regions of HYL1 that are essential for its function in *Arabidopsis thaliana* plant model. We generated mutant forms of the protein that retain their structure but affect its RNA-binding properties. The mutant versions of HYL1 were studied both *in vitro* and *in vivo*, and we were able to identify essential aminoacids/residues for its activity. Remarkably, mutation and even ablation of one of the purportedly main RNA binding determinants does not give rise to any major disturbances in the function of the protein. We studied the function of the mutant forms *in vivo*, establishing a direct correlation between affinity for the pri-miRNA precursors and protein activity.

## Introduction

MicroRNAs are a class of post-transcriptional regulators that negatively regulate the expression of mRNAs through complementary base pairing. They originate in endogenous transcipts that fold into hairpin structures (pri-miRNA). The miRNAs are located in stem-loop structures within the pri-miRNA and are released through the action of RNAse III type enzymes. The resulting ≈21 nt mature molecules are subsequently incorporated into the effector RNA-induced silencing complex (RISC), guiding the complex to target mRNAs through base pair complementarity, resulting in translation inhibition or mRNA degradation [1].

The biogenesis of miRNA in animals proceeds in two steps separated both in time and location. The nuclear RNAse Drosha performs a first cut in the pri-miRNA, releasing the stem loop (pre-miRNA). This RNA molecule is exported to the cytoplasm where Dicer (Dcr) excises the first 22 nt of the pre-miRNA, and this final miRNA molecule is subsequently transferred to the RISC complex. MicroRNA processing in plants differs substantially, as a single RNAse III enzyme, DCL1, produces both of the staggered cuts necessary to release the miRNA from the primary miRNA transcript in the nucleus [2].

A common feature to all the RNA interference (RNAi) processing machineries characterized so far is the participation of accessory RNA binding proteins in the dicing reaction. In animals, Drosha is helped by the double stranded RNA Binding Domain (dsRBD) containing protein DGCR8 (DiGeorge syndrome critical region gene 8), whereas Dicer processing of pre-miRNA requires the presence of TRBP (human immunodeficiency virus transactivating response RNA-binding protein). In *Drosophila melanogaster* while pri-miRNAs are processed by Drosha/PASHA (DGCR8 homologous) in the nucleus and Dcr-1/Loquacious (Loqs) in the cytoplasm, the exogenous double stranded RNA (dsRNA) are generated by Dcr2/Loqs-R2D2. Loqs is required by Dcr-1 for efficient processing of certain classes of pre-miRNAs into mature miRNAs, but it is dispensable for miRNA RISC loading [3], [4]. In contrast, Dcr-2 is able to process dsRNA templates in the absence of R2D2, but requires this double stranded RNA Binding Protein to form the Risc Loading Complex (RLC), which thermodynamically orientates siRNA duplexes onto Ago2 for passenger strand cleavage and active siRNA loaded RISC formation [5], [6]. Dcr-2 also requires the help of the dsRBD containing protein R2D2 for the generation of small interference RNA (siRNA). But the exact molecular role of these dsRBD-containing proteins in RNA processing has not yet been established.

Several accessory proteins that participate in the plant miRNA-processing complex have been identified, most prominently SERRATE (SE) and HYL1 [7], [8]. The protein HYL1 has been shown to be essential for accurate digestion of the miRNA precursors both *in vivo* and *in vitro* [9], [10]. It was also suggested to participate in miRNA strand selection [11]. The sequence of HYL1 contains two dsRBDs in its N-terminus (residues 1–170) followed by a long, presumably unstructured, C-terminal region (residues 171–419) containing six repeats of a 28 aminoacid sequence. The two dsRBDs of the protein are sufficient for its activity in miRNA processing [12].

HYL1 and other helper proteins have similar domain architectures, consisting of two or three dsRBDs organized in tandem. However, the RNA binding properties of these helper proteins is variable. Although R2D2 contains two dsRBDs it does not bind siRNA alone, but requires the presence of Dcr-2 [5], [13], [14]. In TRBP, the first two domains interact with precursor RNA, whereas the third one does not [15]–[17]. In HYL1 the first domain is the one that dominates RNA binding, with the second one interacting only weakly [18], [19]. Substrate binding is supposed to be an essential part of the helper protein function, however it has not been established whether it is an absolute requirement for the participation of these proteins in miRNA biogenesis, and what the consequences of altering the substrate binding affinity of the proteins in the processing mechanism *in vivo* may be. In order to clarify this issue in the present work we sought to identify the RNA binding determinants of HYL1 and to assess their importance on the function of the protein *in vivo*.

## Materials and Methods

### Plant material and growth conditions

*Hyl1-2* (SALK_064863) plants of *Arabidopsis thaliana* (Col-0 ecotype), used for all experiments, were obtained from the Arabidopsis Biological Resource Center (ABRC). Plants were grown on MS medium with 50 µg/ml kanamycin and transplanted to soil at 23°C under long days (16 h light/8 h dark) in a growth room.

### Construction of mutant protein vectors

The cDNA sequence of the HYL1 gene was originally isolated from a mixed cDNA library of *Arabidopsis* by PCR and cloned in pBluescript (pBS) plasmid. A BamHI site at the 5′ end and a SalI site at the 3′ end were introduced for the construction of plant binary vectors. Overlapping PCR, using the cDNA of HYL1 as template, was used to make the five mutants versions of HYL1 (K17A/R19A, K38A, H43A/K44A, Δ40–46 and R67A/K68A. The primers used for cloning and mutagenesis are shown in Text S1. Mutations were verified by DNA sequencing, and the wild-type and mutants versions of HYL1 were introduced into the binary vector CHF5 under the control of the cauliflower mosaic virus 35S.

All binary constructs were transformed into *Agrobacterium tumefaciens*. Genetic transformation of homozygous *hyl1-2* mutant plants was performed using the floral dip method [20]. For selection of transgenic plants, seeds were grown on soil supplemented with 0.2 g/l BASTA at 23°C under long days (16 h light/8 h dark) in a growth room. To classify the phenotype of T1 lines, the size and shape of the rosette leaves were examined. We analyzed 30 to 50 T1 plants of each group.

### Expression analysis

Inflorescence RNA was extracted using TRIzol reagent (Invitrogen) and 0.5 µg of total RNA was treated with RQ1 RNase-free DNAse (Promega). Next, first-strand cDNA synthesis was carried out using SuperScriptTM III Reverse Transcriptase (Invitrogen) with the appropriate primers. PCR reactions were performed in an Mx3000P QPCR System (Stratagene) using SYBR Green I (Roche) to monitor double-stranded (ds) DNA synthesis. Quantitative (q) PCR of each gene was carried out for at least three biological replicates, with technical duplicates for each biological replicate. MiR164a, miR172a and miR396a levels were concurrently determined in each sample by stem-loop RT-qPCR [21]. The relative transcript level was determined for each sample, normalized using PROTEIN PHOSPHATASE 2A cDNA level [22]. Primer sequences are detailed in Text S1.

### Protein expression and purification

Fragments corresponding to the first dsRBD of HYL1 protein (HYL1-1) and their R1, R2 and R3 mutants were amplified by PCR from the binary vectors for plant transformation using the primers shown in Text S1, cloned into the pET-TEV expression vector and sequenced [23]. The plasmids were transformed in *E. coli* BL21 (DE3) cells, which were then grown at 37°C in M9 minimal medium supplemented with 1 g/l ^15^N-NH_4_Cl (Cambridge Isotope Laboratories) in the case of NMR experiments and in LB medium for the stability and affinity experiments.

Protein expression was induced with 1 mM IPTG (isopropyl-β-D-thiogalactopyranoside) at OD_600_ ≈0.7 and cells were grown for 4–5 hours. Cells were harvested, resuspended in a buffer containing 100 mM phosphate, 10 mM Tris, 5 mM β-mercaptoethanol, 8 M Urea pH 8 and disrupted by sonication. The denatured proteins were purified using a Ni (II) column and refolded by dyalisis in 100 volumes of 100 mM phosphate, 50 mM NaCl, 5 mM β-mercaptoethanol, 50 mM glutamate, 50 mM arginine. The refolded proteins were then digested using 1∶100 mass ratio of His-tagged TEV protease, to remove the His-tag, and the protease was removed by a further passage through a Ni (II) column.

### RNA synthesis

RNA samples were produced by *in vitro* transcription with T7 RNA polymerase, using annealed oligonucleotides. Briefly, a mix was prepared containing 1X transcription buffer [40 mM Tris (pH 8), 5 mM DTT, 1 mM spermidine, 0.01% Triton X-100, and 80 mg/mL PEG 8000], each rNTP at 4 mM (rA, rC, rG, and rU), 20 mM MgCl2, 40 µg/mL BSA, and 1 unit of pyrophosphatase, and the annealed template at 35 µg/mL. The reaction was started by addition of T7 RNA polymerase and allowed to proceed for 3 h at 37°C. Then, 50 units of RNase-free DNase were added, and the mix was further incubated for 30 min at 37°C. The reaction mixture was then diluted 8-fold in 20 mM Tris, 10 mM EDTA, and 8 M urea (pH 8.0) and loaded on a Q-Sepharose column equilibrated with the same buffer. The column was eluted with a gradient from 0 to 1 M NaCl in the same buffer. Fractions containing RNA, as determined by A_260_, were checked via denaturing 5% polyacrylamide gel electrophoresis. The fractions with the desired transcript were pooled, dialyzed three times against 200 volumes of H_2_O, and lyophilized for storage before being used.

### Fluorescence anisotropy titrations

For fluorescence anisotropy titrations, RNA fragments were labeled with fluorescein using the 5′ EndTag Nucleic Acid End Labeling System and fluorescein maleimide-thiol reactive label from Vector Laboratories. Labeled fragments were purified by phenol extraction, precipitated with ethanol, and resuspended on 10 mM phosphate buffer (pH 7.0). The double stranded RNA binding partner used is presented in Figure S1. The fluorescence anisotropy was measured on a Varian Cary Eclipse spectrofluorimeter exciting the sample at 492 nm and measuring emission at 520 nm. Anisotropy values were obtained from the average of three measurements with an integration time of 20 s. The excitation and emission slits were set to 10 nm. Labeled RNA was annealed by heating at 100°C for 5 min and chilling at 0°C in an ice-water bath. Data points were fit to the following equation:
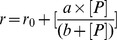



Where *[P]* corresponds to free protein concentration, *r_0_* is the anisotropy of free RNA, *a* is the amplitude of the change in anisotropy upon binding and *b* is the dissociation constant. Titration curves were normalized for plotting by subtracting from each data point the value of *r_0_* and dividing the result by amplitude *a*.

### Protein unfolding experiments by CD

For the stability experiments, the refolded proteins were desalted in PD-10 Desalting Columns (GE Healtcare) using 10 mM phosphate buffer pH 7. Protein concentration was measured by UV spectroscopy. HYL1-1 and HYL1-1 mutants were incubated with urea at different concentrations for three hours at room temperature to ensure equilibrium conditions and to minimize chemical modifications. Ellipticity of protein samples was evaluated using a Jasco 810 spectropolarimeter calibrated with (+) 10-camphorsulphonic acid. Far–UV CD spectra were recorded in the range between 190 and 250 nm, urea induced unfolding was monitored by changes of the ellipticity at 220 nm. Protein concentration was 10 µM, and a cell of 0.1 cm path length was used. In all cases, data were acquired at a scan speed of 20 nm min^−1^ and at least 3 scans were averaged for each sample. The signal at 250 nm was used as an internal control to correct for small fluctuations in the baseline. All measurements were done at 20°C.

### Nuclear Magnetic Resonance (NMR) spectroscopy

NMR spectra were recorded at 298 K. All spectra were processed with NMRPipe [24] and analyzed with CCPNMR [25]. To evaluate the state of folding of the protein constructs, a ^1^H-^15^N SOFAST-HMQC spectrum [26] was acquired on a 600 MHz Bruker spectrometer.

### Protein structural modeling and electrostatic field calculations

A model for the structures of the each of the mutant proteins was generated using the software Rosetta [27]. The electrostatic field of the proteins was calculated with the APBS tool [28], and represented setting 1.5 V isocontours for positive and negative potentials.

## Results

### Rational design of mutations in HYL1

**Figure 1:**
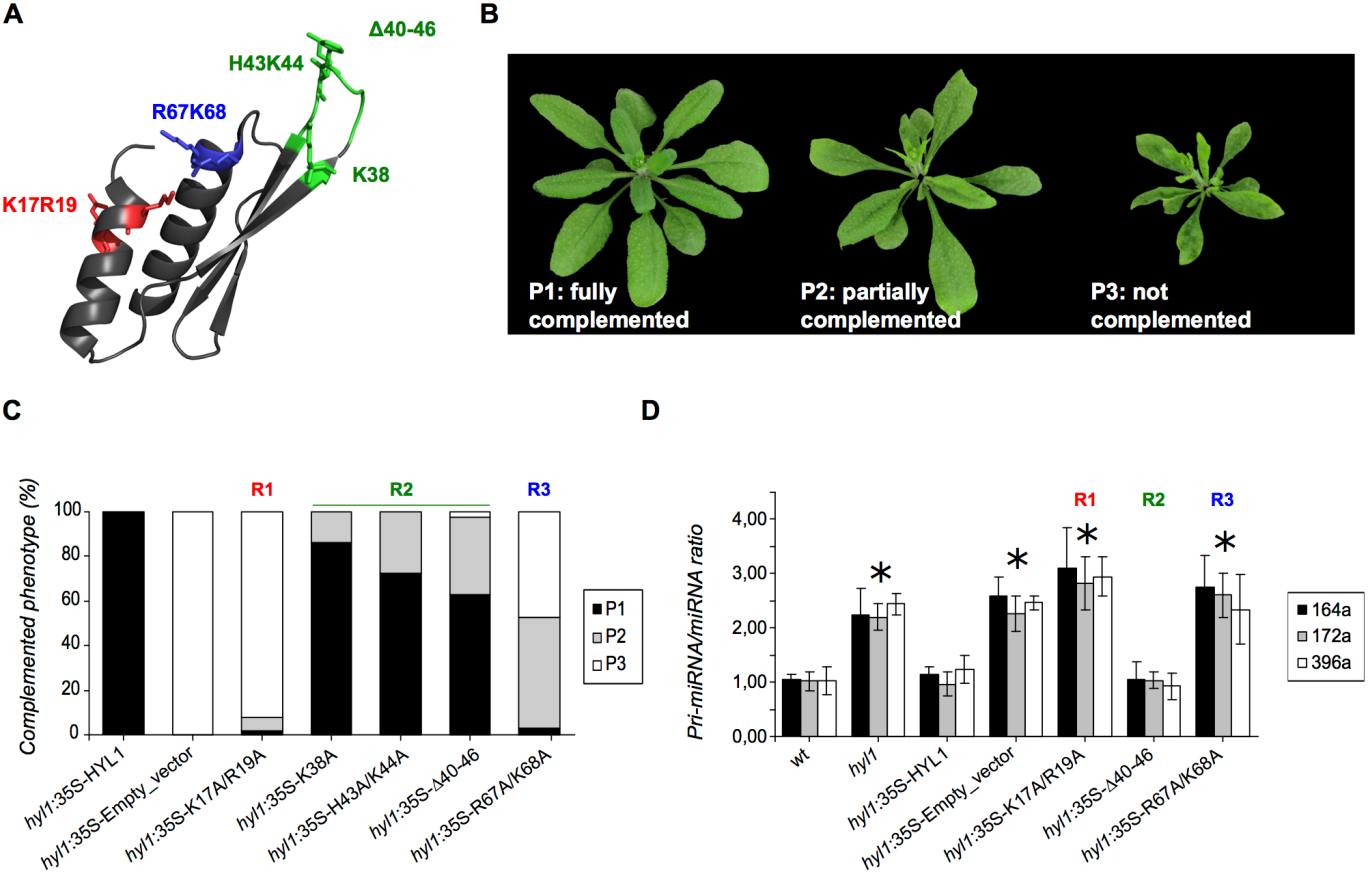
In vivo analysis of HYL1 dsRBD1 mutants. *A. Structure of HYL1-dsRBD1*. The residues that were mutated in the present study are highlighted in colours: region 1, red, region 2, green, region 3, blue. B. *T1 lines phenotypes*. Different phenotypes were found in T1 lines obtained after *hyl1* plants transformation. The different lines included the complete sequence for HYL1 protein with the mutations in the first dsRBD mentioned in Figure 1A. Within each set of transgenic lines, the plants were classified as fully complemented (P1), partially complemented (P2) or not complemented (P3) as function of their rosettes phenotypes. C. *Phenotype analysis of transgenic plants*. The plants were clustered according to each phenotype, as shown in B. The coloured bars represent the fraction of the T1 plants classified as P1 (black), P2 (gray) and P3 (white). D. *Functional analysis of complemented plants.* Relative transcript levels of pri-miRNA164a, pri-miRNA172a and pri-miRNA396a as well as their respective mature miRNAs were determined for each genotype, normalized using *PROTEIN PHOSPHATASE 2A* (*PP2A*, AT1G13320) and compared to WT. The graph shows the ratio of pri-miR164a/miR164a (black), pri-miR172a/miRNA172a (gray) and pri-miRNA396a/miRNA396a (white) levels for each genotype. The plants overexpressing HYL1 with the first dsRBD mutated in regions 1 and 3 (R1 and R3) presented less pri-miRNA processing efficiency compared to plants overexpressing HYL1 with the Δ40–46 deletion in region 2 (R2). Data shown are mean ± SEM of 3 biological replicates. Asterisks indicate significant differences between genotypes, as determined by ANOVA (*P<0.001).

The function of HYL1 in plants is defined by its two N-terminal double stranded RNA binding domains [12]. However, we and others have shown that both domains have different roles within the protein [18], [19]. While the first domain binds tightly to substrate RNA, the second one shows little affinity for the same primary miRNA transcripts and contributes to a small extent to the overall RNA-binding affinity of the protein. Therefore, it can be concluded that the substrate recognition function of HYL1 is located in the first dsRBD. In order to understand the structural determinants of this recognition, we introduced mutations in the regions of this domain that interact with RNA. Crystal and solution structures of several dsRBDs in complex with RNA show that these domains bind dsRNA via the sidechains of three separated regions of the protein. A group of well-conserved residues in helix 1 (region 1) and the loop between strands 1 and 2 (region 2) interact with the OH moieties of backbone riboses, whereas a set of basic residues, either Arg or Lys, located at the N-terminus of helix 2 (region 3) interact with the phosphate backbone [29]–[33]. Our NMR characterization of the interaction between HYL1 and dsRBD1 showed a similar pattern of interactions [18]. Therefore, we decided to probe the relevance of each region to the function of the protein by the introduction of mutations based on the NMR results. With this aim we constructed a double mutant in region 1 (K17A/R19A), one point mutant, one double mutant and a deletion of the whole loop mutant in region 2 (K38A, H43A/K44A and Δ40–46) and a double mutant in region 3 (R67A/K68A) (Figure 1A). We considered that these different variants would bring new insights into the role of the different domains of HYL1 both *in vivo* and *in vitro*.

### *In* *vivo* function of HYL1 variants

In order to verify the relevance of the changes introduced in HYL1-dsRBD1 for the function of the protein *in vivo* we employed a complementation experiment with the *hyl1-2* null mutant of *Arabidopsis thaliana* [34]. These plants exhibit a pleiotropic phenotype characterized by hyponastic leaves, reduced leaf size, slow growth, reduced plant height, late flowering and reduced fertility, as well as multiple lateral shoots [35]. In *hyl1-2* plants miRNA processing is impaired, leading to a decrease of mature miRNAs and an increase in the steady state levels of pri-miRNAs [7], [9], [10].

We transformed the mutant plants with the full-length *hyl1* genes bearing the designed mutations, under the control of the 35 S promoter of the *Cauliflower mosaic virus*. The first generation of transgenic plants show varying phenotypic features that range between strong *hyl-1* phenotype to fully complemented wild type phenotype. We classified the plants as completely rescued (P1), partially rescued (P2) or not rescued (P3), based on their phenotypic characteristics (Figure 1B). A distribution of phenotypes among the primary transgenic plants of *Arabidopsis* is expected, as the transgenes insert at random position along the genome, and are then influenced in different ways by the genomic environment. This has been previously observed for transgenes in general and also for different domains of HYL1 in particular [12]. The classification shows that the level of phenotypic rescue cluster within regions and that, unexpectedly, the highest deficiency in HYL1 function corresponds to mutants in regions 1 and 3, whereas mutants in region 2 show high levels of phenotypic rescue, indicating that the region is mostly unimportant for the function of HYL1 *in vivo* (Figure 1C). This last result was puzzling, since several previous studies on homologous proteins have shown that this loop is essential for dsRNA recognition by dsRBDs [36], [37]. With the aim of further testing this result, we produced a mutant containing a deletion of the whole loop corresponding to region 2 (Δ40–46). This mutant protein resulted functional as well, thus confirming that region 2 is dispensable for the function of HYL1 (Figure 1C).

Aside from phenotype, it is well established that the function of HYL1 is to assist DCL1 in miRNA processing [9], [10]. We decided to test the function in a direct way by measuring the levels of mature miRNA and primary miRNA transcripts in the transgenic plant lines. In order to correct for possible variations of the expression levels we measured the protein expression level of different plant lines by means of western blot analysis (Figure S2) and selected lines with similar HYL1 levels for further analysis.

We quantified miR164a, miR172a and miR396a together with their corresponding pri-miRNAs in the wild type, *hyl1-2*, and *hyl1-2* mutants transformed with the different constructs (Figure 1D). In all cases, the processing activity corresponds well with the phenotypic rescue. Therefore we can confirm that the mutations in regions 1 and 3 affect the function of HYL1 during pri-miRNA processing.

### Structural analysis of the mutant domains

**Figure 2:**
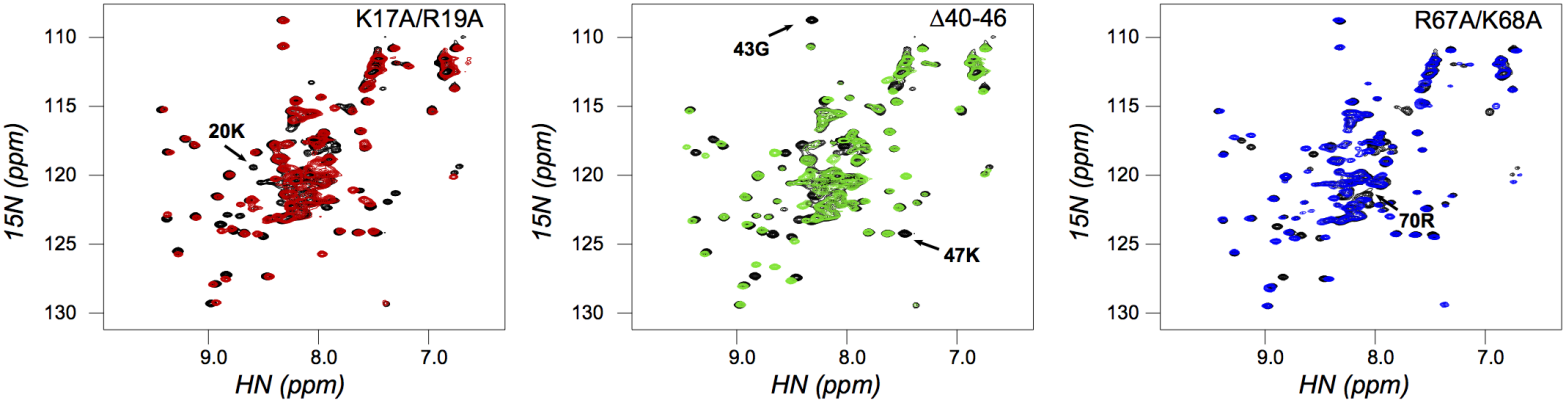
Folding states of HYL1-dsRBD1 mutant domains. SOFAST^ 1^H-^15^N HMQC spectra of the mutant proteins compared to the wild type protein (black in all spectra). The mutant protein K17A/R19A is shown in red, Δ40–46 in green and R67A/K68A in blue. Signals corresponding to the mutated residues are shown with an arrow on the wild type spectra. The mutated domains in region 1, 2 and 3 retain the same folding as the wild type HYL1 dsRBD1.

The introduction of mutations can lead to either local or global changes in the structure of the protein. We were interested in verifying the involvement of the mutated sidechains in the function of HYL1. Therefore, with the aim of ruling out large structural rearrangements that would invalidate our results, we generated constructs for the isolated dsRBD1, labeled the proteins with ^15^N and obtained a ^1^H-^15^N HMQC spectrum of each of the mutants. This kind of spectrum is exquisitely sensitive to alterations in the structure of the protein, and allows for the localization of these alterations with residue definition. The superposition of the spectra of the different mutants with that of the wild type protein shows that the structural alterations brought by the mutations are mostly restricted to the regions structurally close to the mutations themselves, not leading to global modifications (Figure 2). This is true even for the deletion mutant, where one could expect a more important rearrangement of the protein structure.

**Figure 3:**
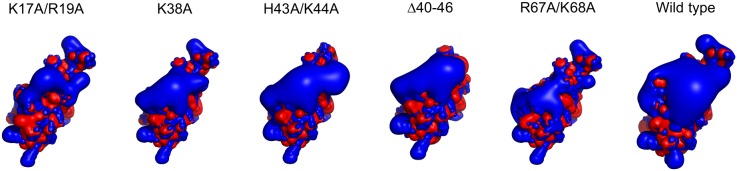
Electrostatic field calculations on the wild type and mutant proteins. The electrostatic field around each modeled protein and HYL1-dsRBD1 wild type was calculated using APBS [28]. The 1.5 V isocontours are shown for positive (blue) and negative (red) potential. Mutants in regions 1 and 3 display a big disruption of the positive electrostatic patch in the dsRNA-binding surface for substrate binding.

In order to find a structural basis for the alterations in HYL1 function brought about by the mutations, we obtained structural models of the mutant proteins based on the crystal structure of the wild type protein (3ADG.pdb) using the software Rosetta [27]. As expected, the point mutations do not lead to large structural rearrangements, and the deletion of the β1–β2 loop is well-tolerated by the protein fold (Figure S3). Overall, the more important difference in the HYL1 variants resides in the electrostatic potential field around the protein. The wild type protein has a large positive electrostatic potential patch around regions 1 and 3, caused by a cluster of basic residues. These residues contact the phosphate backbone of the RNA via electrostatic interactions. Removal of these basic side chains in region 1 and region 3 mutants, lead to a highly reduced electrostatic potential. In contrast, the mutants in region 2 show a mostly conserved electrostatic potential (Figure 3). Therefore, we conclude that the electrostatic attraction of the substrate by residues in regions 1 and 3 of HYL1 is essential for the correct function of the protein.

### Stability and substrate binding

**Figure 4:**
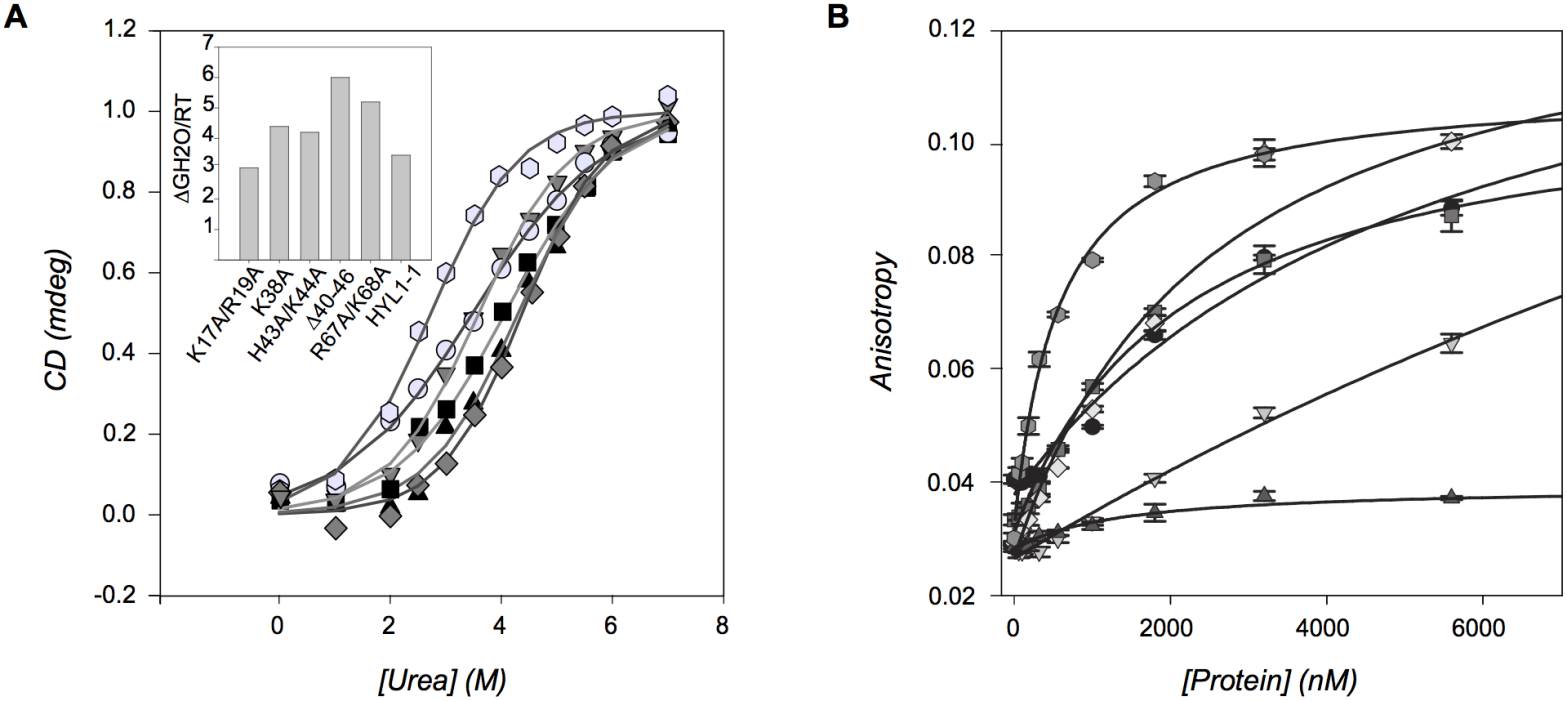
Biophysical characterization of HYL1-dsRBD1 mutant domains. A. *Protein stability measurements*. Induced urea unfolding of the wild type and mutant proteins were followed by circular dichroism at 220 nm. In the left, on the top, the ΔGH_2_O/RT of each protein is shown. Mutants in region 2 and 3 display an increase of the stability relative to wild type, whereas the mutant in region 1 and the wild type have comparable values. B. *Substrate binding affinity of the mutant domains*. Fluorescence anisotropy of labeled substrate RNA was measured at increasing protein concentration. All the mutant proteins have lower binding affinities than the wild type domain. The mutants in region 1 and 3 have the most reduced binding affinities. In A and B symbols correspond to the following proteins: gray hexagons, wild type; black triangles, K17A/R19A; gray circles, Δ40–46; gray diamonds, K38A; black squares H43A/K44A; gray triangles R67A/K68A.

Having established that all the mutant proteins were correctly folded, we wondered if the differences observed on *in vivo* activities could be due to a destabilization of the protein induced by the mutations introduced. Therefore we measured the stability (ΔG°N-U) of the HYL1 dsRBD1 and the corresponding mutants. For this purpose, we used circular dichroism to monitor urea-induced denaturation. We observed that protein stability is actually increased in mutants in region 2 and 3 relative to wild type, whereas the stability of the mutant in region 1 is comparable to that of the wild type protein (Figure 4A). From these results we could conclude that mutations do not impair HYL1 dsRBD1 stability. In order to evaluate the effect of the mutations on the binding affinity for substrate RNA we resorted to a fluorescence polarization assay, using the isolated dsRBD1 constructs and the lower-stem region of pri-miR172 as binding partner. All mutant proteins showed a decrease in binding affinity. Most noticeably, the double mutant in region 1 (K17A/R19A) hardly bound RNA, even at the highest protein concentration tested, whereas the double mutant in region 3 (R67A/K68A) showed a much-reduced affinity (Figure 4B).

Quite strikingly, considering the purported importance of region 2 in binding, all mutations in this region gave only slightly diminished binding affinities. The dissociation constants of these mutants are similar, ranging between 2.5 and 5.4 µM, giving an overall coherent picture of the influence of this region in HYL1 affinity for RNA. Even when the whole loop is deleted, the protein retains significant affinity for the substrate.

## Discussion

The exact function of HYL1 within the plant miRNA-processing complex is not understood at present. The protein is known to be important for the accuracy of the processing of miRNA, as it was demonstrated both *in vitro* and *in vivo* [9], [10]. In fact, recent works have shown that the lack of HYL1 can be compensated with a more active form of DCL1 [38], [39]. In this case, enough correctly processed miRNA can be generated, thereby limiting the effects of inaccuracy. HYL1 was also suggested to participate in miRNA strand selection and delivery to AGO1 within the RISC complex, much in the same way as TRBP in humans [11]. While there has been more effort in establishing the role of similar dsRBD-containing helper proteins in other organisms, there has been no consensus on their function so far.

Although the mechanism of HYL1 function in miRNA processing is not fully understood, binding to substrate RNA seems to be an essential part of it. RNA binding by HYL1 is dominated by dsRBD1, with marginal contribution to the binding affinity by dsRBD2 and the rest of the protein [18], [19]. In the present paper we dissected the function of each of the dsRNA binding determinants within HYL1 dsRBD1.

Our characterization shows that regions 1 and 3 of the domain are the most important structural determinants of RNA binding. In most dsRBDs, region 3 contains a well-conserved KKxAK motif that recognizes the phosphodiester backbone of the dsRNA major groove. These residues form a significant electrostatic patch on the surface of the domain created by the positively charged lysine side chains (Figure 3). In HYL1, however, the motif is split between region 1 and region 3: the third lysine residue in the motif is replaced by a glutamate, but this substitution is compensated by the presence of a lysine residue in position 17, whose terminal amino group is located in a position equivalent to that of the lysine absent in region 3. The mutations that we introduced in regions 1 and 3 give rise then to a structurally similar result, that is, the disruption of this electrostatic patch. In this way we can rationalize the similar impact on both function and affinity of both mutations introduced. It was suggested that electrostatic interactions play an important role in dsRBD-RNA recognition [37], [40] although these effects seem to be dissimilar between different dsRBDs [41]. The absence of alterations in stability on the mutant proteins that could hinder RNA recognition highlight that phosphate backbone recognition is essential for RNA binding by these domains.

An unexpected result in our work is that the loop β1–β2 in dsRBD1 is dispensable for HYL1 activity and has little influence on the RNA-binding affinity. This result is difficult to rationalize from a structural point of view, as the loop plays an important role in RNA binding in other reported cases, inserting in the dsRNA minor groove. It provides with a set of direct interactions with the ribose moieties and the bases, and contributes to the recognition of the of the A-form RNA double helix, as it is located exactly two turns away from the region 1 interaction position. The importance of this loop in other dsRBDs has been demonstrated through mutational analysis [36], [37]. However, in HYL1 the highly conserved histidine residue at the top of the loop can be mutated and even the whole loop deleted without major changes in RNA affinity, protein stability or protein function. This shows that the importance of the loop in RNA recognition can be dissimilar among dsRBDs. In this respect, it is noteworthy that the dsRBDs of Dicer proteins have a short loop that could not in principle participate in RNA binding, or at least not if the dsRBD binds RNA in the canonical way [42], [43]. The absence of the loop was suggested to hinder RNA binding by these domains [43], but it was recently shown that Dicer dsRBD do bind dsRNA and miRNA precursors [44]. This experimental evidence goes in line with our results, showing that the loop β1–β2 is dispensable for dsRNA binding by HYL1. A structural study of the complex formed by these domains or by the HYL1 deletion mutant with RNA would be necessary to understand how the binding mode of these proteins differs from that of canonical dsRBDs. The regulation of HYL1 activity by phosphorylation was also recently demonstrated [45]. Remarkably, one of the regulatory phosphorylation sites is S42, located within this loop. When this serine residue is mutated to aspartic acid, mimicking phosphorylation, the function of the protein is inhibited, whereas when the phosphorylation sites are eliminated by mutation of serines to alanines the resulting protein is fully functional. Considering such a fact, we can speculate that mutations in region 2 could hinder a natural inhibition of HYL1 activity by phosphorylation, therefore offsetting the partial loss of affinity introduced by the mutation.

In summary, we could show that there is a direct correlation between substrate binding affinity by HYL1-dsRBD1 and its *in vivo* activity. Our work also establishes that RNA binding by HYL1 dsRBD1 is essential for its function. These results contribute to understanding the participation of this protein in substrate recognition within the plant miRNA processing machinery.

## Supporting Information

Figure S1 **Structure of pre-miRNA 172-ls.** The pre-miRNA 172 ls was used to measure the binding affinities of the wild type and mutated dsRBD1-HYL1 proteins. The double stranded RNA was labelled in the 5′ end with fluorescein (see materials and method section).
(DOCX)

Figure S2 **HYL1 expression level in inflorescences.** A. The phenotype rescue of hyl1 plants do not depend to the HYL1 expression level. Transgenic plants with similar HYL1 levels are complemented in different ways and they clustered in different groups (see figure 1B). B. The expression levels of the inflorescences of 36 T1 lines were determined. The amount of recombinant HYL1 protein are highly variables. We selected 3 plants with similar protein levels to continue with further studies (e.i miRNA processing efficiency). The numbers after each label indicate the plant ID. The wells where the bands are absent indicate that protein levels are under the detection limit. The film exposure time was 2 minutes.
(DOCX)

Figure S3 **Modeled structure of HYL1-dsRBD1.** Hyl-dsRBD1 Δ40–46 (left), compared to wild type HYL1-dsRBD1 (PDB 3ADG, right). The structure of the HYL1 mutant was modeled using Rosetta [27]. The final structure adopt a folding that is similar to the crystallographic structure of the wild type HYL1 dsRBD.
(DOC)

Text S1 **Methods and primers information.** Western blot method and primer sequences information.
(DOCX)
