# Supplementary material for: Structural Determinants of Arabidopsis thaliana Hyponastic Leaves 1 Function In Vivo
Source: PLoS One. 2014 Nov 19;9(11):e113243. doi: 10.1371/journal.pone.0113243 (PMC4237382; doi:10.1371/journal.pone.0113243)

1. *Figure S1*

Structure of pre-miRNA 172-ls

The pre-miRNA 172 ls was used to measure the binding affinities of the wild type and mutated dsRBD1-HYL1 proteins. The double stranded RNA was labelled in the 5´ end with fluorescein (see materials and method section).


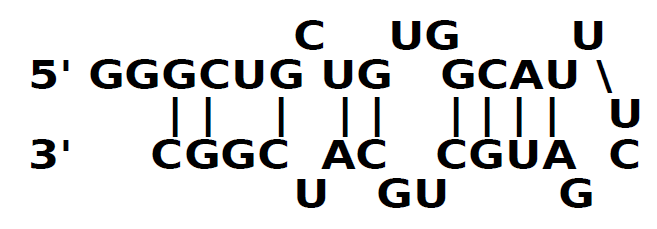

Supplement: Figure S1 — Structure of pre-miRNA 172-ls. The pre-miRNA 172 ls was used to measure the binding affinities of the wild type and mutated dsRBD1-HYL1 proteins. The double stranded RNA was labelled in the 5′ end with fluorescein (see materials and method section). (DOCX) [file pone.0113243.s001.docx]
