# Supplementary material for: Structural Determinants of Arabidopsis thaliana Hyponastic Leaves 1 Function In Vivo
Source: PLoS One. 2014 Nov 19;9(11):e113243. doi: 10.1371/journal.pone.0113243 (PMC4237382; doi:10.1371/journal.pone.0113243)
Supplement: Figure S2 — HYL1 expression level in inflorescences. A. The phenotype rescue of hyl1 plants do not depend to the HYL1 expression level. Transgenic plants with similar HYL1 levels are complemented in different ways and they clustered in different groups (see figure 1B). B. The expression levels of the inflorescences of 36 T1 lines were determined. The amount of recombinant HYL1 protein are highly variables. We selected 3 plants with similar protein levels to continue with further studies (e.i miRNA processing efficiency). The numbers after each label indicate the plant ID. The wells where the bands are absent indicate that protein levels are under the detection limit. The film exposure time was 2 minutes. (DOCX) [file pone.0113243.s002.docx]

1. *Figure S2*
   1. HYL1 expression level in inflorescences

A. The phenotype rescue of hyl1 plants do not depend to the HYL1 expression level. Transgenic plants with similar HYL1 levels are complemented in different ways and they clustered in different groups (see figure 1B).

B. The expression levels of the inflorescences of 36 T1 lines were determined. The amount of recombinant HYL1 protein are highly variables. We selected 3 plants with similar protein levels to continue with further studies (e.i miRNA processing efficiency). The numbers after each label indicate the plant ID. The wells where the bands are absent indicate that protein levels are under the detection limit. The film exposure time was 2 minutes.

A)


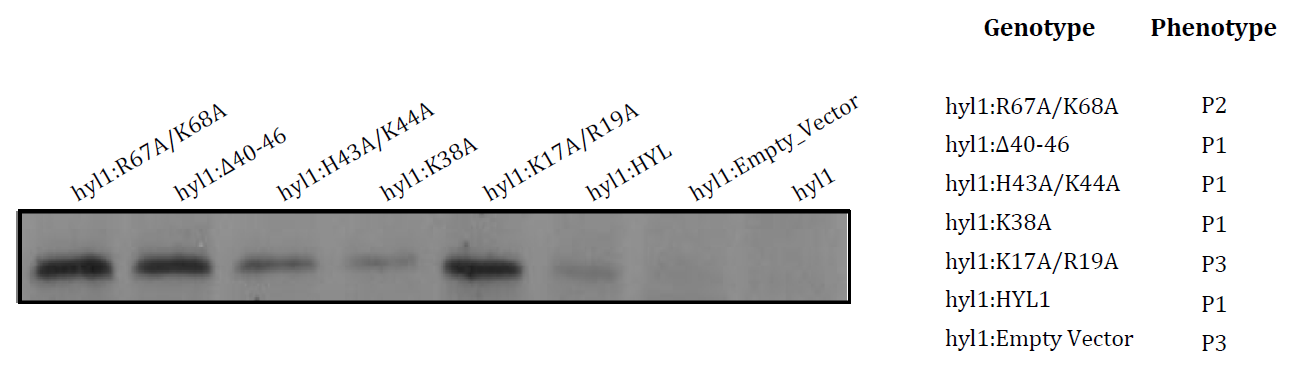


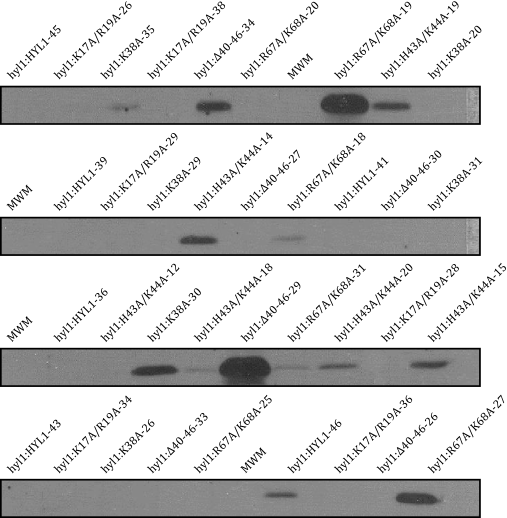
B)
