# Supplementary material for: Structural Determinants of Arabidopsis thaliana Hyponastic Leaves 1 Function In Vivo
Source: PLoS One. 2014 Nov 19;9(11):e113243. doi: 10.1371/journal.pone.0113243 (PMC4237382; doi:10.1371/journal.pone.0113243)

### ***Figure S3.***

***Modeled structure of HYL1-dsRBD1***

Hyl-dsRBD1 ∆40-46 (left), compared to wild type HYL1-dsRBD1 (PDB 3ADG, right). The structure of the HYL1 mutant was modeled using Rosetta [27]. The final structure adopt a folding that is similar to the crystallographic structure of the wild type HYL1 dsRBD.


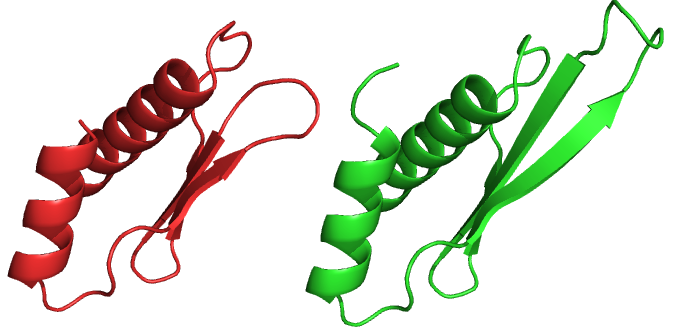

Supplement: Figure S3 — Modeled structure of HYL1-dsRBD1. Hyl-dsRBD1 Δ40–46 (left), compared to wild type HYL1-dsRBD1 (PDB 3ADG, right). The structure of the HYL1 mutant was modeled using Rosetta [27]. The final structure adopt a folding that is similar to the crystallographic structure of the wild type HYL1 dsRBD. (DOC) [file pone.0113243.s003.doc]
