# Supplementary material for: Structural Determinants of Arabidopsis thaliana Hyponastic Leaves 1 Function In Vivo
Source: PLoS One. 2014 Nov 19;9(11):e113243. doi: 10.1371/journal.pone.0113243 (PMC4237382; doi:10.1371/journal.pone.0113243)
Supplement: Text S1 — Methods and primers information. Western blot method and primer sequences information. (DOCX) [file pone.0113243.s004.docx]

# Text S1

- - 1. *Samples collection method*

Closed inflorescences were collected in 2 ml tubes from 2 months aged plants. The samples were maintained in liquid nitrogen and the storage at -80 °C until the usage.

- - 1. *Protein extraction for Western Blot analysis*

The inflorescence tissues were homogenized in liquid nitrogen and 5 ul of buffer solution were added per 1 mg of tissue (50 mM Tris-Hcl pH 7.8, 1 mM MgCl_2_ 2 mM EDTA (Ethylenediaminetetraacetic acid), 1 mM PMSF (phenylmethylsulfonyl fluoride), 2 mg/ml Ascorbate and 0.3 mg/ml reduced glutathione. The homogenates were centrifuged at 13,000 g, 4 °C for 30 minutes. Finally the supernatant was transferred to a clean tube. Protein concentration were determined using bovine serum albumin as standard protein and Bradford reagent [1].

- - 1. *Method for Western Blot analysis*

1. For blotting analysis, 40 µg of protein were resolved on 12 % SDS-PAGE and transferred to nitrocellulose membrane (Amershan biosciences). After blocking with 5% Albumin in 0.1% Tween 20 PBS (137 mM NaCl, 10 mM Phosphate, 2.7 mM KCl, 0.1 %) blots were incubated overnight with anti-Hemaglutinin (anti-HA) antibody (1:1000 Sigma).
2. The membranes were then incubated with horseradish peroxidase-conjugated secondary antibody and developed using a chemiluminiscence detection kit (Amersham biosciences).
   - 1. *Primers used in the quantitation of pri-miRNA and miRNA*

For reverse transcription: Oligo dT: TTT TTT TTT TTT TTT TTT TTT TTT V

Stem loop oligo (SLO) for miR164a: GTCTCCTCTGGTGCAGGGTCCGAGGTATTCGCACcagaggagACYGCACG

*SLO for miR396a:* GTCTCCTCTGGTGCAGGGTCCGAGGTATTCGCACcagaggagACMAGTTC

*SLO for miR172a:* GTCTCCTCTGGTGCAGGGTCCGAGGTATTCGCACcagaggagACMTGCAG

For PCR:

*PP2A:*

Fw: CCTGCGGTAATAACTGCATCT

Rv: CTTCACTTAGCTCCACCAAGCA

*miRNA164a:*

Fw: GGCGGTGGAGAAGCAGGGCA

Rv: TGGTGCAGGGTCCGAGGTATT

*miRNA172a:*

Fw: ggcggAGAATCTTGATGATG

Rv: TGGTGCAGGGTCCGAGGTATT

*mIRNA396a:*

Fw: GGCGGTTCCACAGCTTTCTT

Rv: TGGTGCAGGGTCCGAGGTATT

*miR164a precursor:*

Fw: GCGGAGCTCTGCTTGGAAATGCGGGTGAGAATCTCC

Rv: CGCGGATCCTATATAACATCAATGGGTGAAGAGCTC

*miRNA172 precursor:*

Fw: GCGGAGCTCCCGGAGCCACGGTCGTTGTTGGCTG

Rv: CGCGGATCCGGAAAGAATAGTCGTTGATTGCCG

*miRNA396a precursor:*

Fw: CCTGGATCCGTATTCTTCCACAGCTTTCTTGAAC

Rv: CCTCTGCAGTGTATCTTCCCACAGCTTTATTGAAC

- - 1. *Primers for the introduction of mutations in HYL1*

HYL1_BamHI FW: GCGGGATCCATGACCTCCACTGATGTTTCCTC

HYL1_SalI RV: GCGGTCGACTGCGTGGCTTGCTTCTGTCTCC

HYL1_K17A/R19A FW: CCAATTGCTATGTTTTCgcAAGTgcgTTGCAGGAGTATGCTC

HYL1_K17A/R19A RV: GAGCATACTCCTGCAAcgcACTTgcGAAAACATAGCAATTGG

HYL1_K38A FW: CCTGTTTATGAGATCGTTgcAGAAGGCCCTTCAC

HYL1_K38A FW: GTGAAGGGCCTTCTgcAACGATCTCATAAACAGG

HYL1_H43A/K44A: FWCGTTAAAGAAGGCCCTTCAgcCgcATCTTTATTTCAATCG

HYL1_H43A/K44A RV: CGATTGAAATAAAGATgcGgcTGAAGGGCCTTCTTTAACG

HYL1_∆40-46 FW: AAGAAtctggaTTATTTCAATCGACTGTGATACTGG

HYL1_∆40-46 RV: AATAAtccagaTTCTTTAACGATCTCATAAACAGG

HYL1_R67A/K68A FW: GCCTGGATTCTTCAATgcagcGGCTGCAGAGCAATCAGC

HYL1_R67A/K68A RV: GCTGATTGCTCTGCAGCCgctgcATTGAAGAATCCAGGC

- - 1. *Primers for subcloning HYL1-dsRBD1 in the expression vector*

HYL1-dsRBD1 FW: ggaattccatatgATGACCTCCACTGATGTTTCCTC

HYL1-dsRBD1 RV: cggatccTCATCCCGTTTCGTGAACAGGTTGTGA

- 1. **Supporting references**

[1]. Bradford, M. M. A rapid and sensitive method for the quantitation of microgram quantities of protein utilizing the principle of protein-dye binding. *Anal. Biochem. 72***,** 248–54 (1976).
